# TolCV1 inhibition by NPPB renders *Vibrio vulnificus* less virulent and more susceptible to antibiotics

**DOI:** 10.1128/aac.00502-24

**Published:** 2024-12-13

**Authors:** Yue Gong, Rui Jiang, Rui Hong Guo, Se Jin Jo, Hyeongju Jeong, Kyuho Moon, Joon Haeng Rhee, Young Ran Kim

**Affiliations:** 1 College of Pharmacy and Research Institute of Pharmaceutical Sciences, Chonnam National University 501645 , Gwangju, Republic of Korea; 2 Department of Pharmacy, The First Affiliated Hospital of Zhengzhou University 191599 , Zhengzhou, China; 3 College of Pharmacy, Kyung Hee University, Seoul, Republic of Korea; 4 Clinical Vaccine R&D Center and Department of Microbiology, Combinatorial Tumor Immunotherapy MRC, Chonnam National University Medical School 501645 , Hwasun-gun, Jeonnam, Republic of Korea; Columbia University Irving Medical Center, New York, New York, USA

**Keywords:** NPPB, TolCV1, *Vibrio vulnificus*, antimicrobial susceptibility, RtxA1 secretion

## Abstract

Bacterial efflux pumps play important roles in the antibiotic resistance and excretion of virulence factors. We previously characterized that TolCV1, a component of efflux pumps, plays critical roles in resistance to antibiotics and bile and also RtxA1 toxin secretion of *Vibrio vulnificus*. In this context, we speculated that TolCV1 blockers would have a dual effect of enhancing susceptibility to antibiotics and suppressing virulence of *V. vulnificus*. Here, we show that the chloride channel blocker 5-nitro-2-(3-phenylpropylamino) benzoic acid (NPPB) increases susceptibility to antibiotics and suppresses cytotoxicity of *V. vulnificus* through inhibition of TolCV1. NPPB significantly decreased TolCV1 in *V. vulnificus* cells by liberating the protein from the cell body. Checkerboard assay showed that NPPB enhanced the antimicrobial activities of antibiotics such as kanamycin, tetracycline, erythromycin, and ampicillin against *V. vulnificus*. Moreover, NPPB inhibited the secretion of RtxA1 toxin and protected host cells from *V. vulnificus*-induced cytotoxicity. In addition, NPPB markedly suppressed *V. vulnificus* growth in the presence of bile salts and enhanced the therapeutic effect of tetracycline in *V. vulnificus*-infected mice. The safety and efficacy of NPPB were confirmed at the cellular and animal levels. Collectively, TolCV1 inhibition by NPPB renders *V. vulnificus* less virulent and more susceptible to antibiotics.

## INTRODUCTION

*Vibrio vulnificus* is a Gram-negative halophilic estuarine pathogen causing fatal septicemia and severe wound infections (1). *V. vulnificus* is a highly fatal human pathogen with a high mortality rate (~50%) (2). Currently, *V. vulnificus* is categorized into three biotypes based on biochemical characteristics. Biotype 1 is typically associated with infections in humans, which cause the majority of ingestion cases (primary septicemias) and most wound infections. Biotype 2 strains are primarily found in farmed eels and rarely in humans (2). Biotype 3 causes human wound infections, and they have mostly been reported among tilapia aquaculture workers in Israel (3). Considering the high mortality rate, antibiotics are immediately prescribed to patients diagnosed with possible *V. vulnificus* infections (4). However, *V. vulnificus* strains isolated from environmental or clinical specimens exhibit multi-antibiotic resistance (5), and the rapid emergence of antibiotic-resistant strains may cause serious public health and economic concerns (6). In this regard, effective treatment of *V. vulnificus* infections must be complemented by targeted antimicrobial stewardship strategies to address and mitigate the development of antibiotic resistance.

In Gram-negative bacteria, tripartite efflux pumps are operating to export various toxic substances and metabolites (7). Tripartite efflux pumps contribute to antimicrobial resistance by decreasing effective drug concentration in the cytoplasm (8). TolC, an outer membrane protein involved in the assembly of various tripartite efflux pumps, is embedded in the outer membrane and forms a continuous channel to extrude toxic substances out of bacteria (9). Two TolC homologs (TolCV1 and TolCV2) were identified in *V. vulnificus* (10). We previously investigated the functions of TolCV1 and TolCV2 in *V. vulnificus* (11) and found that TolCV1 plays key roles in the pathogenesis of *V. vulnificus* by affecting antibiotic resistance, RtxA1 toxin secretion, bile resistance, and mice lethality. These findings suggested that TolCV1 could serve as a plausible target for the development of antibiotic-enhancing drugs against *V. vulnificus* infections.

Various inhibitors of TolC-dependent efflux pumps have been identified, including phenylalanyl-arginyl-β-naphthylamide (PAβN), sodium malonate, MBX2319, BDM88855, and BDM91288. PAβN is a well-known broad-spectrum inhibitor of efflux pumps in Gram-negative bacteria, such as the AcrAB-TolC, MexAB−OprM, MexCD−OprJ, MexEF−OprN, and AdeABC. PAβN was able to potentiate the activity of different families of antibiotics such as fluoroquinolones, macrolides, oxazolidinones, chloramphenicol, and rifampin but not aminoglycosides (12, 13). In addition, PAβN significantly reduced biofilm formation and increased bacterial outer membrane permeability (14, 15). Sodium malonate, a natural, nonhazardous, small molecule, is a newly discovered efflux pump inhibitor that showed a significant inhibitory effect on AcrAB-TolC and other multidrug efflux pumps in *Escherichia coli* (16). MBX2319, a pyranopyridine efflux pump inhibitor, showed a potent inhibitory effect against RND efflux pumps of the *Enterobacteriaceae* (17). BDM88855 and BDM91288 are pyridylpiperazine-based AcrB efflux pump inhibitors, which can potentiate the activity of antibiotics against *E. coli* and *Klebsiella pneumoniae* (18, 19).

The roles of efflux pump protein TolC in antibiotic resistance and RTX toxin secretion have significant implications for the development of efficacious therapeutic modalities against *V. vulnificus* infections. Through extensive screening for regulators of efflux pumps, we discovered that 5-nitro-2-(3-phenylpropylamino) benzoic acid (NPPB) substantially decreases TolCV1 protein level in *V. vulnificus* cells. NPPB is a nonspecific chloride channel blocker that has been proven to have antiglaucoma (20), anticancer (21, 22), anti-atherosclerosis (23), and anxiolytic (24) effects. The systemic injection of NPPB rendered rat to exhibit an anxiolytic profile through astrocyte inactivation and GABA release inhibition (24). Many investigations have evaluated the efficacy and safety of NPPB in various human cell lines (25, 26) and mouse models (27–30), providing support that the drug is reasonably safe for use in initial, small-scale clinical studies. In this study, we evaluated the protective effects of NPPB on *V. vulnificus*-induced mice lethality in combination with antibiotics.

## MATERIALS AND METHODS

### Bacteria, cell cultures, and reagents

*V. vulnificus* strains were cultured in LB broth (Becton-Dickinson, MD, USA) at 200 rpm in a 37°C incubator. *V. vulnificus* MO6/24-O is a clinical isolate (31), while CMM744 is MO6/24-O with a deletion mutation in *rtxA1* gene (*rtxA1-*) (32). The *tolCV1* mutant is MO6/24-O with a deletion mutation in *tolCV1* gene (*tolCV1-*) (11). HeLa cells (Korean Cell Line Bank, Seoul, Korean) were cultured in Dulbecco’s Modified Eagle’s medium (DMEM; Welgene, Daegu, Korea) containing 10% fetal bovine serum (FBS; ThermoFisher Scientific, Waltham, MA, USA) in a 37°C incubator with 5% CO_2_. NPPB was acquired from Sigma-Aldrich (St. Louis, MO, USA) and dissolved in dimethyl sulfoxide (DMSO, Sigma-Aldrich) to prepare a 100 mM stock solution. The stock solution was filter-sterilized and stored at −20°C in the dark.

### Western blotting

*V. vulnificus* strains were cultured overnight and diluted 200-fold with fresh LB broth for another 4 h. In total 1 × 10^9^ colony-forming units (CFU) of bacterial cells were cultured in LB broth supplemented with or without NPPB (12.5, 25, 50, and 100 µM) for 1 h. Later, the optical density of cultures was measured with a Biophotometer (Eppendorf, Germany) at 600 nm (OD_600_). Then, 2 × 10^8^ CFU of bacterial cells were counted and used for Western blotting. The culture supernatants (300 µL) were precipitated by adding threefold volumes of cold acetone. The precipitations were resuspended in SDS-PAGE sample buffer and boiled at 100°C for 10 min. The proteins were electrophoresed on 10% SDS-PAGE gels, transferred onto PVDF membranes (Millipore Ltd, Tullagreen, Carrigtwohill, Germany), and blocked with 5% skim milk. The membranes were then incubated with appropriate primary antibodies against TolCV1 (1:500) (33), TolCV2 (1:2000) (11), and OmpU (1:1000) (34). Followed by probing with horseradish peroxidase-conjugated secondary antibodies. Immunoreactive bands were visualized with the WesternBright ECL-Spray (Advansta, Menlo Park, CA, USA), as previously described (35). To detect RtxA1 protein levels, NuPAGE 3-8% tris-acetate gradient gels (Invitrogen, Carlsbad, CA, USA) and an anti-RtxA1 primary antibody specific to amino acids 1492-1970 (RtxA1-D2, 1:250) were used as previously described (36).

### Determination of the binding of TolCV1 and NPPB

#### Co-immunoprecipitation assay

*V. vulnificus* cells (1.5 × 10^10^ CFU) cultured in LB broth for 4 h were treated with NPPB (50 µM) in DPBS (Welgene, South Korea) for 1 h. After centrifugation, the bacterial pellets were collected and incubated in RIPA buffer with anti-TolCV1 antibody (1:50) at 4℃ overnight on a rocker. Subsequently, the mixture was incubated with protein A/G PLUS-Agarose (Santa Cruz Biotechnology, Inc., USA) with gentle shaking at 4°C for 3 h. The mixture was centrifuged at 2,500 rpm for 5 min and washed with RIPA buffer (Thermo Fisher Scientific, USA) four times. The immunoprecipitates resuspended in water were boiled for 10 min, and the supernatants were subjected to Western blotting to determine the amount of TolCV1 protein.

#### Liquid chromatography-mass spectrometry for NPPB detection

The presence of NPPB in the immunoprecipitates captured by anti-TolCV1 was analyzed using an Agilent Technologies 1260 Series Infinity II LC system (Agilent Technologies, Santa Clara, CA, USA), coupled with an Agilent G6125B MSD system (Agilent Technologies, Santa Clara, CA, USA). A Phenomenex Luna reversed phase C18 column (100 × 4.6 mm, 5 µm) was used in this experiment. Data analysis was performed based on the UV spectra and mass values after injecting the samples into the liquid chromatograph-mass spectrometer (LC-MS). The mobile phase was controlled under a linear gradient condition: 50% MeCN to 100% MeCN in H_2_O with 0.1% formic acid over 20 min, followed by 100% MeCN after 20 min at a flow rate of 0.4 mL/min. The mass spectrometer was operated in the electrospray ionization positive ion mode using a scan mode with a capillary voltage of 3 kV, a drying gas flow of 12 L/min, a nebulizer pressure of 35 psig, and a drying gas temperature of 350℃.

### Construction of a tolCV1 point mutant strain (P4) with alanine substitutions of four key amino acids (300, 301, 303, 305), the potential binding sites between *V. vulnificus* TolCV1 and NPPB

The chromosomal *V. vulnificus tolCV1* point mutant strain was constructed using PCR-based mutagenesis and suicide plasmid pDM4. In order to construct a PCR template for the site-directed mutagenesis, a DNA fragment containing *tolCV1* gene and upstream flanking gene was amplified from *V. vulnificus* MO6-24/O chromosomal DNA (accession number NC_014966.1) using the primer pairs (*tolCV1*-F: 5′-GGACTAGTGCACCTCACTGAGTGAATCTTGTAG-3′; *tolCV1*-R: 3′-CCGCTCGAGCGATTACTTTTTCGCAATCAAACC-5′), and cloned into the suicide plasmid pDM4, generating pDM4::TolCV1. Site-directed mutagenesis was performed using a Phusion Site-Directed Mutagenesis kit (Thermo Fisher Scientific Baltics UAB, V.A., Lithuania) in accordance with the manufacturer’s protocol. Alanine substitutions of four key amino acids (300, 301, 303, 305) on *tolCV1* gene were generated by PCR using the primers with desired mutations (F300A-N301A-G303A-N305A-F: 5′-GCCGCTGTGGCTGTAGCTCTCGTTGTTCCACTCTATACCG-3′; F300A-N301A-G303A-N305A-R: 5′-AGCTACAGCCACAGCGGCATTATTGGTTGTACC

ATCACTTTGTGC-3′). The colonies with successful substitutions were further confirmed by DNA sequencing (Macrogen Inc., Korea). The resulting pDM4 with desired substitutions on *tolCV1* gene was transformed into *E. coli* SY327λpir, and then *E. coli* SM10λpir, which provides a trans-acting conjugative function for the plasmid. The pDM4 with desired substitutions on *tolCV1* gene (pDM4::*tolCV1*△P4) was introduced into *V. vulnificus* chromosome by conjugation of pDM4::*tolCV1*△P4 in *E. coli* SM10 *λpir* with *V. vulnificus* MO6-24/O recipient cells. Stable CM^R^ transconjugants, which contain the plasmid integrated into the chromosome by first homologous recombination, were selected on thiosulfate-citrate-bile salt sucrose (TCBS) agar plates containing chloramphenicol. Subsequently, chloramphenicol-resistant colonies were cultured on 2.5% NaCl HI agar plate with 10% sucrose. The individual colonies were screened for the loss of pDM4 on 2.5% NaCl HI agar plates and 2.5% NaCl HI agar plates with chloramphenicol. As a result of second homologous recombination, the vector sequence is excised out leaving only wild-type or *tolCV1* point mutant strains. Finally, the positive colonies with *tolCV1* point mutations were verified via DNA sequencing.

The complementation of P4 mutant was constructed using previous constructed plasmid pLAFR3::*tolCV1* (33), which was introduced into P4 mutant strain by triparental mating. Stable transconjugants were selected and confirmed by PCR and Western blot analysis.

### Effect of tolCV1 point mutation (P4) on *V. vulnificus*-induced cytotoxicity

*V. vulnificus* wt, P4, and *tolCV1*-mediated cytotoxicity was detected by a CytoTox96 nonradioactive cytotoxicity assay kit (Promega, Madison, WI, USA). HeLa cells were seeded in 48-well plates (5 × 10^4^ cells/well, SPL Life Sciences Co., Ltd., Pocheon-si, Korea) in DMEM containing 10% FBS and incubated overnight. After washing with serum-free DMEM twice, the cells were pretreated with NPPB at 12.5, 25, 50, and 100 µM for 1 h and then infected with *V. vulnificus* strains at the multiplicity of infection (MOI) of 100 for 90 min. Lactate dehydrogenase (LDH) in the supernatants was analyzed as a marker of cytotoxicity (37).

### Effect of tolCV1 point mutation (P4) on *V. vulnificus* resistance to bile salt

We tested the effect of *tolCV1* point mutation (P4) on *V. vulnificus* resistance to bile salt. Overnight cultures of *V. vulnificus* wild-type, *tolCV1* deletion mutant, or *tolCV1* point mutant (P4) strains were diluted 200-fold with fresh LB media in the presence or absence of 0.02% bile salt. Bacterial cells were cultured in a shaking incubator at 37°C, and the growth was measured using a spectrophotometer at 600 nm every 2 h. Results are representative of at least three independent experiments.

To test the survival of *V. vulnificus* strains on thiosulphate-citrate-bile salt sucrose agar plate, 3 μL of overnight cultures were dropped on the agar plates and then incubated overnight at 37°C.

### Checkerboard assay

The combination effects of NPPB and antibiotics were assessed by the checkerboard assay as previously described (12). Checkerboards were set up with twofold serial dilutions of NPPB (ranging from 12.5 to 800 µM) and antibiotics. The *V. vulnificus* culture grown overnight was diluted to approximately 1 × 10^6^ CFU/mL and then added to 96-well plates. Plates were read at 630 nm using a microplate reader (BioTek, Winooski, VT, USA) after 24 h of incubation at 37°C. The combination effect was assessed by calculating the fractional inhibitory concentration index (FICI) (12). The FICI was defined as synergistic (FICI ≤0.5), additive (0.5 < FICI ≤ 1), irrelevant (1 < FICI ≤ 4), or antagonistic (FICI >4).

### Real-time PCR

Overnight cultured *V. vulnificus*, *tolCV1-*, and P4 mutant strains were diluted 200-fold and incubated for 4 h with fresh HI broth. Total RNAs were extracted from the bacterial cells using TRI reagent (Molecular Research Center, Inc., USA). The transcriptional activities of *recA*, *rtxA1*, and *tolCV1* were evaluated using real-time PCR (qPCR) with the primer pairs (*recA*-F: 5′-GAC CAG TTG TTG GTA TCT CAG CC-3′; *recA*-R: 5′-CGA TTT CTG CCT TTG GCG TCA-3′), (*rtxA1*-F:5′-CTG AAT ATG AGT GGG TGA CCT ACG-3′; *rtxA1*-R: 5′-TGC GGT TTG ATT TCA CCG C-3′), and (*tolCV1*-F: 5′-AGC AAA ACG ATC CAC AGT TAT TGA-3′; *tolCV1*-R: 5′-ACG TTC AGG TTT TTA TGC TCT TGA-3′), respectively (33). The qPCR was performed following the protocol outlined previously (35). The *recA* gene was used as an internal control for qPCR, and the relative mRNA expression levels of *rtxA1* and *tolCV1* were normalized to *recA* levels using the threshold cycling (∆∆C_T_) method.

### Effect of NPPB on *V. vulnificus*-induced cytotoxicity

*V. vulnificus*-mediated cytotoxicity was detected by a CytoTox96 nonradioactive cytotoxicity assay kit (Promega, Madison, WI, USA). HeLa cells were seeded in 48-well plates (5 × 10^4^ cells/well, SPL Life Sciences Co., Ltd., Pocheon-si, Korea) in DMEM containing 10% FBS and incubated overnight. After washing with serum-free DMEM twice, the cells were treated with NPPB (12.5, 25, 50, and 100 µM) for 1 h before *V. vulnificus* infection at the multiplicity of infection of 100 for 90 min. Lactate dehydrogenase in the supernatants was analyzed as a marker of cytotoxicity (37).

### Effect of NPPB on host cell growth

Host cell viability was detected using the MTS (Promega) assay. HeLa cells seeded in 96-well plates (1 × 10^4^ cells/well, SPL Life Sciences Co., Ltd.) were treated with NPPB (12.5, 25, 50, and 100 µM) for 24 h and then incubated with 3-(4,5-dimethylthiazol-2-yl)-5-(3-carboxymethoxyphenyl)-2-(4-sulfophenyl)−2H-tetrazolium (MTS) according to the manufacturer’s instructions. The absorbance at 490 nm was recorded using a microplate reader (BioTek, Winooski, VT, USA).

### Staining of HeLa cells infected with *V. vulnificus*

HeLa cells were seeded in 8-well-chambered coverslips of German borosilicate (Nalge Nunc International, Rochester, NY, USA) overnight in DMEM containing 10% FBS. After washing with serum-free DMEM, the cells were treated with NPPB (100 µM) for 1 h before *V. vulnificus* infection at an MOI of 100 for 90 min. Cells were washed with Hank’s Balanced Salt Solution (HBSS; Welgene Inc., Daegu, South Korea) and then stained with red-fluorescence Alexa Fluor 594 wheat germ agglutinin (WGA) for plasma membrane and Hoechst 33342 dye for nucleus (Molecular Probes, Image-iT LIVE plasma membrane and nuclear labeling kit #I34406). The images were captured using a fluorescence microscope (Nikon DS-Ri2 microscope camera, Tokyo, Japan).

### Combined effect of bile salt and NPPB on the growth of *V. vulnificus*

*V. vulnificus* strains were incubated in LB broth at 200 rpm in a 37°C incubator overnight and diluted 1,000-fold with fresh LB broth. The diluted suspensions were inoculated into 96-well microplates with or without 50 µM NPPB or/and 0.02% bile salt. The plates were cultured at 37°C, and absorbance was measured at 630 nm by a microplate reader (BioTek, Winooski, VT, USA) every 2 h.

To count the CFUs of live bacteria at each time point, the cultures were 10-fold serially diluted with phosphate-buffered saline (PBS; Himedia, India). Then the different dilutions (10 µL) were dropped on LB agar plates and incubated overnight at room temperature. The numbers of the grown colonies were counted.

### Mouse lethality test

All animal procedures were performed following the guidelines of the Animal Care and Use Committee of the Chonnam National University, and the protocol was approved by the committee (Approval Number: CNU IACUC-YB-2020-82). Healthy 8-week-old CD-1 female mice were purchased from Damool Science (Daejeon, Korea) and housed in pathogen-free animal facilities under a 12-h light/dark cycle at room temperature with a humidity of 55 ± 5%. The mice were caged randomly in four groups of 10 mice each. Overnight cultured *V. vulnificus* strains were diluted 200-fold with fresh LB broth and grown for 4 h to reach log-phase. *V. vulnificus* log-phase cells (2 × 10^6^ CFU/mouse) were administered to the mice via the intraperitoneal injection. One day or 2 h before *V. vulnificus* infection, mice were pretreated with tetracycline (0.75 mg/kg) or NPPB (4 or 20 mg/kg) via intraperitoneal injection twice. The control group infected with wild-type was pretreated with the same amount of PBS. The infected mice were observed in the animal room for 48 h.

### Safety of NPPB on mouse splenocytes

BALB/c female (7 weeks) mice were sacrificed via cervical dislocation. From the isolated mouse spleen, a single-cell suspension was prepared using a mesh strainer. After removing red blood cells, mouse splenocytes were seeded in 96-well plates and treated with NPPB at different concentrations for 72 h. Cell viability was assayed using MTS reagent according to the manufacturer’s protocols. Absorbance at 490 nm was measured using an enzyme-linked immunosorbent assay microplate reader (ELx808, BioTek Instruments, Inc.).

### Safety of NPPB on mouse liver and kidney

To evaluate the toxicity of NPPB in ICR female (8 weeks) mice, NPPB was administered intraperitoneally for 3 days. At the end of the experiment, mouse serum was collected from the heart and analyzed using a serological analyzer (Fujifilm Corp.). The serum aspartate transaminase (AST) and alanine transaminase (ALT) levels were determined to evaluate liver function, whereas the serum creatinine (CRE) and blood urea nitrogen (BUN) levels were determined to evaluate kidney function.

### Statistical analysis

All results were presented as means ± SEM. Statistical significance was evaluated by GraphPad Prism, version 5.01 (San Diego, CA, USA), and one-way ANOVA followed by a Tukey post hoc test, where *P* < 0.05 was considered statistically significant. Each experiment was performed at least in triplicates and three biological replicates. The results shown were from representative experiments.

## RESULTS

### NPPB decreased TolCV1 protein level in *V. vulnificus* cells

**Fig 1:**
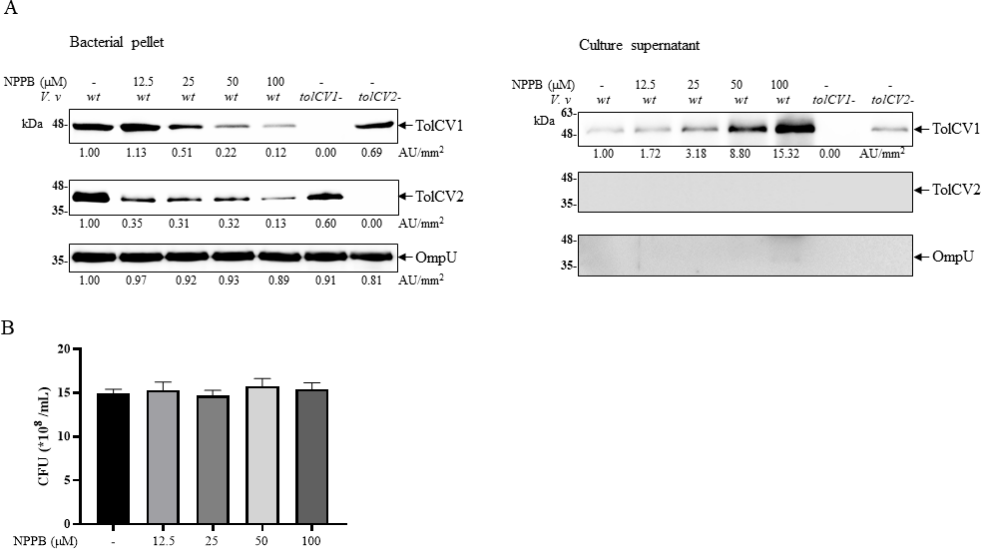
Effect of NPPB on TolCV1 protein. (**A**) Overnight cultured *V. vulnificus* cells were diluted 200-fold with fresh LB broth for 4 h. Bacteria (1 × 10^9^ CFU/mL) were pretreated with NPPB for 1 h. Bacterial pellets (2 × 10^8^ CFU) and proteins in supernatant (300 µL) were analyzed by Western blotting with anti-TolCV1 and anti-OmpU antibodies. Proteins were quantified with ImageJ software. (**B**) The optical density of cultures after treatment with NPPB for 1 h was measured using a Biophotometer at 600 nm. Abbreviations: *V.v*, *V. vulnificus*; wt, wild-type; tolCV1-, *tolCV1* mutant strain of MO6-24/O; *tolCV2*-, *tolCV2* mutant strain of MO6-24/O.

To delineate the effect of NPPB on TolCV1, we checked the level of the efflux pump protein in supernatants and bacterial pellets. NPPB lowered TolCV1 and TolCV2 protein levels in the bacterial pellet in a dose-dependent manner, whereas an outer membrane protein OmpU was not affected (Fig. 1A). Interestingly, TolCV1 protein was increased in the supernatant by the treatment with NPPB (Fig. 1A). However, TolCV2 and OmpU proteins did not appear in the supernatant of *V. vulnificus* strains. The growth of *V. vulnificus* was not affected by the NPPB treatment (Fig. 1B). This result suggests that NPPB rather specifically interacts with TolCV1 and depletes from the outer membrane to the supernatant, which may compromise the efflux of antibiotics.

### NPPB directly binds with TolCV1

**Fig 2:**
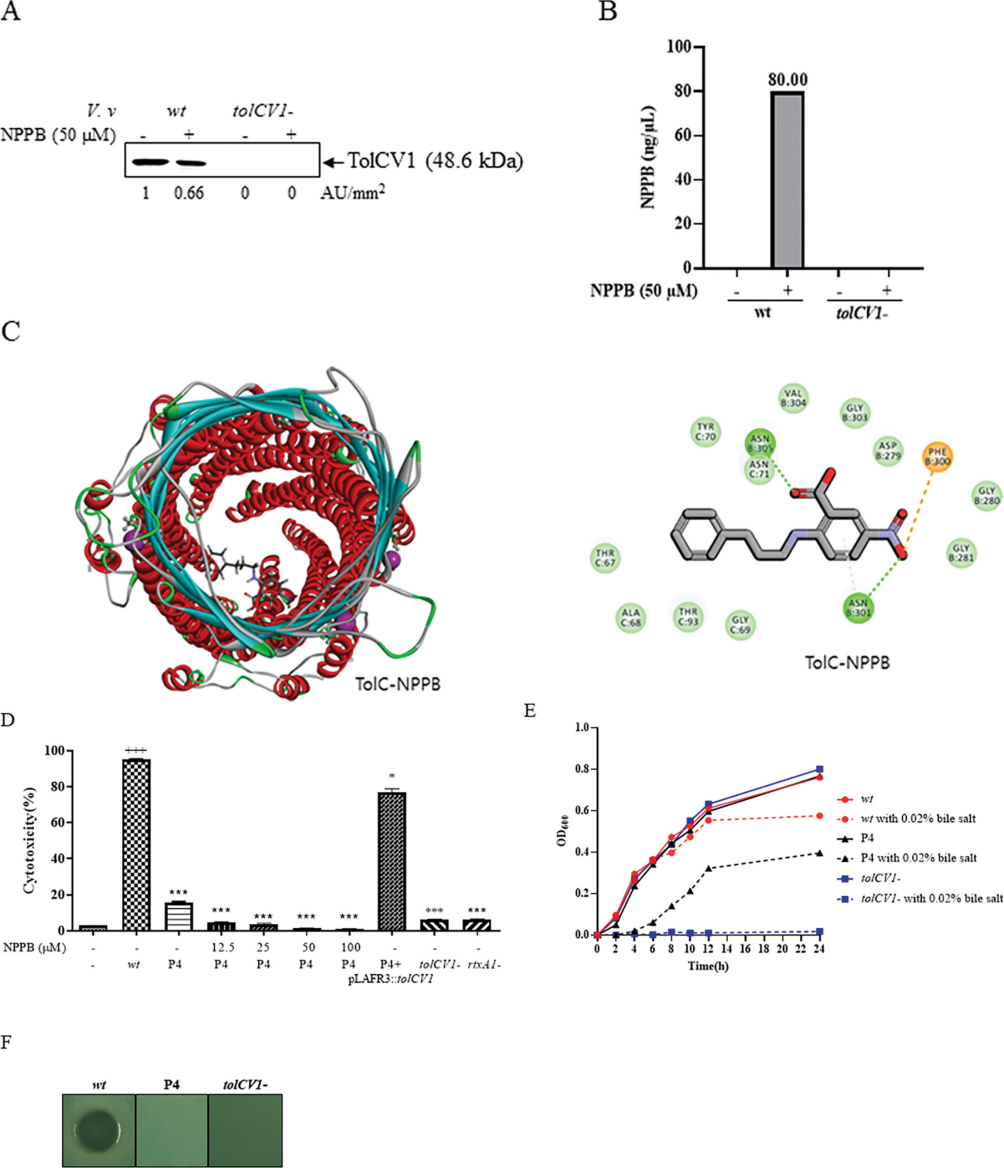
NPPB directly binds with TolCV1 protein of *V. vulnificus*. (**A**) The amount of TolCV1 protein in immunoprecipitates of *V. vulnificus* wt and *tolCV1*- strains was measured by Western blotting with anti-TolCV1 antibody. (**B**) The amount of NPPB in the immunoprecipitates captured by anti-TolCV1 antibody was analyzed by using LC-MS analysis. (**C**) Molecular docking study to predict the scoring and binding interactions between NPPB and *V. vulnificus* TolCV1. Some residues, namely, TYR C:70, ASN C:71, VAL B:304, GLY B:303, ASP B:279, GLY B:280, GLY B:281, GLY C:69, THR C:67, THR C:93, and ALA C:68 were thought to participate in this interaction. By a closer look at the obtained results, it is clear that NPPB forms hydrogen bonds with these residues (ASN B:301, ASN B:305, PHE B:300). (**D**) HeLa cells were treated with NPPB for 1 h and then infected with *V. vulnificus* strains at an MOI of 100 for 90 min. LDH in the supernatants was measured as a marker of cytotoxicity (^***^P < 0.001 vs *V. vulnificus* wild-type; ^*^P < 0.05 vs *V. vulnificus* wild-type; ^+++^*P* < .001 vs untreated control). (**E**) Overnight cultured *V. vulnificus* wt, P4, and *tolCV1*- strains were diluted 200-fold with fresh LB media in the presence or absence of 0.02% bile salt. Bacterial cells were cultured in a shaking incubator at 37°C, and the growth was measured using a spectrophotometer at 600 nm every 2 h. (**F**) Three microliters of overnight cultures of *V. vulnificus* wt, P4, and *tolCV1*- strains were dropped on TCBS agar and then incubated overnight at 37°C. Abbreviations: Mock, untreated HeLa cells; wt, wild-type; P4, *tolCV1* point mutant with four mutations (F300A, N301A, G303A, and N305A); P4+pLAFR3::*tolCV1*, complementary strain of P4 mutant strain *tolCV1*-, *tolCV1* in-frame deletion mutant strain of MO6-24/O; *rtxA1*-, *rtxA1* mutant strain of MO6-24/O.

To identify whether NPPB could directly bind with TolCV1 protein, we performed the immunoprecipitation using TolCV1 antibody for NPPB-treated *V. vulnificus* and then LC/MS analysis to detect the amount of NPPB bound to the protein. NPPB decreased TolCV1 level in *V. vulnificus* wild-type (Fig. 2A; Fig. S1). NPPB in a concentration of 80 ng/µL was detected in the immunoprecipitates of *V. vulnificus* wild-type, not in *V. vulnificus tolCV1* mutant (Fig. 2B), suggesting that NPPB could directly bind with TolCV1 protein of *V. vulnificus*.

Molecular docking study was carried out to simulate the binding interactions between NPPB and *V. vulnificus* TolCV1. The template structure of *V. vulnificus* TolCV1 was downloaded from Protein Data Bank (PDB ID: 1TQQ as *E. coli* TolC). The structure model of *V. vulnificus* TolCV1 was performed using Modeller v9.19. Docking NPPB into the putative binding site of *V. vulnificus* TolCV1 was performed using CDOCKER in Discovery studio, version 4.1, from BIOVIA (San Diego, USA). The potential binding sites in the *V. vulnificus* TolCV1 barrel structure were predicted by the grid-based cavity prediction algorithm of the CDOCKER program. Some residues, namely, TYR C:70, ASN C:71, VAL B:304, GLY B:303, ASP B:279, GLY B:280, GLY B:281, GLY C:69, THR C:67, THR C:93, and ALA C:68, were thought to participate in this interaction. By a closer look at the obtained results, it was assumed that NPPB forms hydrogen bonds with these residues (ASN B:301, ASN B:305, PHE B:300) (Fig. 2C). To address these amino acids’ critical role for TolCV1 in interacting with NPPB, we constructed a *tolCV1* point mutant strain (P4) with four alanine substitution mutations (F300A, N301A, G303A, and N305A), the potential binding sites of *V. vulnificus* TolCV1 and NPPB. *V. vulnificus* rapidly kills host cells, and the RtxA1 toxin is the major player mediating the cytotoxicity (32, 38). As we previously reported, TolCV1 affects the cytotoxicity of *V. vulnificus* by controlling the secretion of RtxA1 (11). We performed an LDH release experiment to observe whether the point mutation (P4) of *V. vulnificus tolCV1* inhibits the cytotoxicity of *V. vulnificus*.

As shown in Fig. 2D, *V*. *vulnificus*-induced cytotoxicity was significantly decreased in a dose-dependent manner in the *tolCV1* point mutant strain (P4), and the defect was restored by an in *trans* complementation, suggesting these four amino acids play critical roles for TolCV1 to export RtxA1 toxin. Subsequently, we monitored the growth rates of wild-type, P4 mutant, and *tolCV1* mutant strains in LB broth containing 0.02% bile salts. The results showed that the growth of the *tolCV1* mutant was completely abolished and the growth of the P4 mutant was also inhibited in the presence of bile salts (Fig. 2E). Moreover, both P4 mutant and *tolCV1* mutant strains showed growth defects on TCBS agar plates (Fig. 2F). Based on these results, we concluded that these four amino acids of TolCV1 play critical roles for TolCV1 to export bile salts and achieve *V. vulnificus* survival in the host intestine. The diverse effects of NPPB on *V. vulnificus*, including RtxA1 toxin secretion, potentiating antibiotic efficacy, and bile resistance, may be attributed to its interaction with those amino acid residues located in the inside of *V. vulnificus* TolCV1 barrel structure and thus impairs the export of these substrates out of bacteria.

### NPPB enhanced the antibacterial effect of antibiotics against *V. vulnificus*

**Fig 3:**
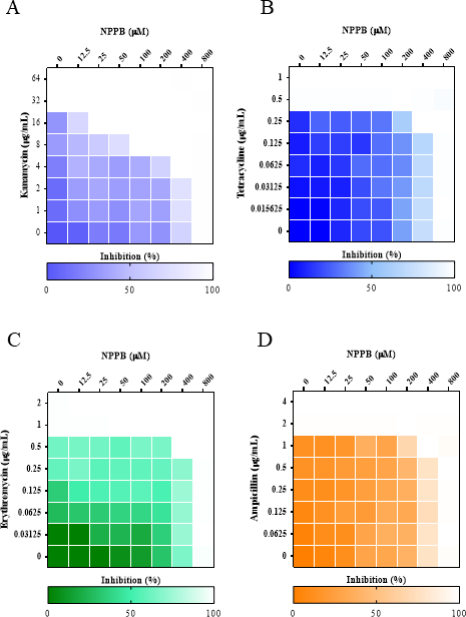
Heat plots of checkerboard assays of antibiotics in combination with NPPB against *V. vulnificus*. (**A**) Kanamycin, (**B**) tetracycline, (**C**) erythromycin, (**D**) ampicillin.

TolCV1 participates in the assemblies of various tripartite efflux pumps; thus, the protein plays a crucial role in the efflux of antibiotics in *V. vulnificus* (39). As reported, the deletion of *tolCV1* rendered *V. vulnificus* more susceptible to chemical detergents, intercalating agents, and antibiotics as compared to wild-type strain (10). Due to the decreased TolCV1 level in bacterial pellets caused by the NPPB treatment, we hypothesized that NPPB may potentiate activities of antimicrobial agents against *V. vulnificus*. Checkerboard assay was conducted to examine the combined antimicrobial activities of NPPB with antibiotics against *V. vulnificus*. The addition of NPPB enhanced the antibacterial effects of kanamycin, tetracycline, erythromycin, and ampicillin against *V. vulnificus* (Fig. 3; Table 1).

**TABLE 1:** Antimicrobial activities of NPPB and antibiotics against *V. vulnificus^a^*

	MIC_Ab_ (µg/mL)		MIC_NPPB_ (µM)		Outcome (FICI)
	Alone	Combination	Alone	Combination	
Kanamycin	32	8	800	25	Synergistic (0.281)
Tetracycline	0.5	0.25	800	400	Additive (1)
Erythromycin	1.0	0.5	800	400	Additive (1)
Ampicillin	2.0	1.0	800	400	Additive (1)

^*a*^ MIC_Ab_, minimal inhibitory concentration of antibiotics against *V. vulnificus* when used alone or in combination with NPPB; MIC_NPPB_, minimal inhibitory concentration of NPPB against *V. vulnificus* when used alone or in combination with antibiotics.

### NPPB suppressed the secretion of RtxA1 toxin

**Fig 4:**
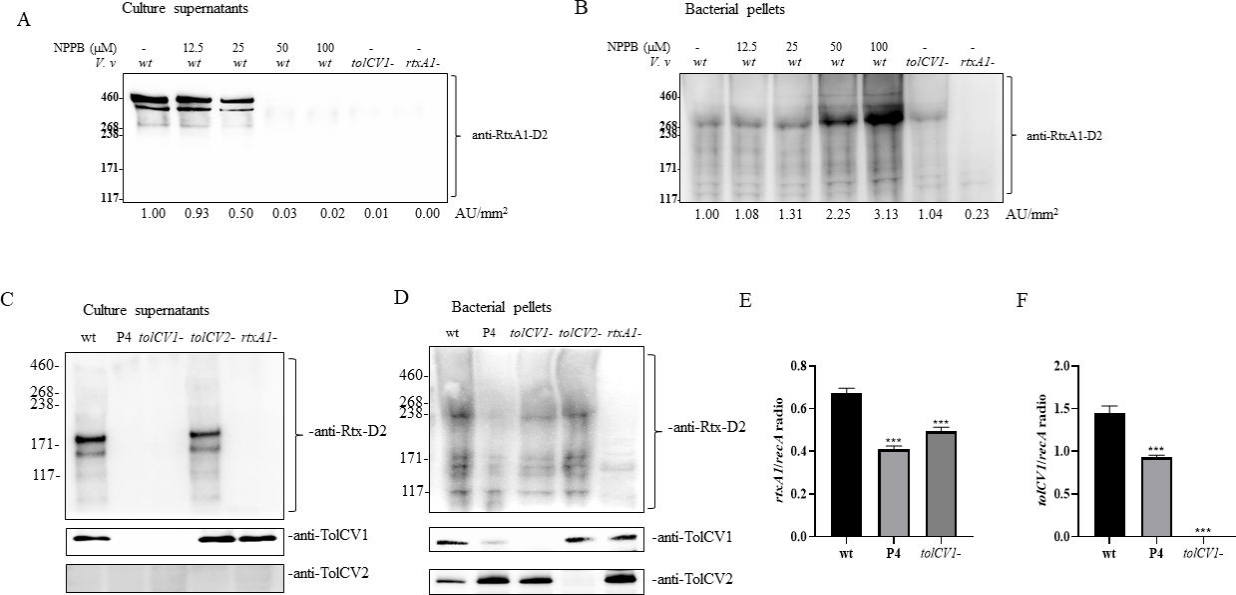
Effect of NPPB on the secretion of RtxA1 toxin in LB broth. (**A–B**) Prepared log-phase bacteria (1 × 10^9^ CFU/mL) were pretreated with NPPB for 1 h. (**C–D**) Overnight cultures of each *V. vulnificus* strains were diluted 200-fold with fresh LB broth and cultured in a 37°C shaking incubator for 4 h. (**A,C**) Proteins in the supernatants were precipitated by cold acetone. (**B,D**) Bacterial pellets (2 × 10^8^ CFU) were prepared. Western blotting was performed with antibodies specific to RtxA1-D2, TolCV1, and TolCV2. Proteins were quantified with ImageJ software. (**E–F**) The transcriptional activities of (**E**) *rtxA1* and (**F**) *tolCV1* were measured by qPCR analysis. The results shown are representative of three independent experiments. Abbreviations: *V.v*, *V. vulnificus*; wt, wild-type; P4, *tolCV1* point mutant with four mutations (F300A, N301A, G303A, and N305A); *tolCV1*-, *tolCV1* mutant strain of MO6-24/O; *tolCV2*-, *tolCV2* mutant strain of MO6-24/O; *rtxA1*-, *rtxA1* mutant strain of MO6-24/O.

Among the cytotoxic substances produced by *V. vulnificus*, RtxA1 dominantly contributes to the necrotic host cell death and lethality in mice (38, 40, 41). We previously reported that RtxA1 toxin was exported through TolCV1-dependent efflux systems of *V. vulnificus* (11). In this context, we examined the levels of RtxA1 toxin in culture supernatant and bacterial pellets by Western blotting using an anti-RtxA1 antibody (40). NPPB treatment increased RtxA1 remaining in bacterial pellets in a dose-dependent manner (Fig. 4B), while it decreased RtxA1 protein in the culture supernatants (Fig. 4A). We examined the effect of *tolCV1* point mutation (P4) on the secretion and expression of RtxA1, TolCV1, and TolCV2 by Western blotting. The proteins of TolCV1 and RtxA1 were significantly reduced in the culture supernatant and bacterial pellet of P4 mutant. However, P4 mutation did not show any inhibitory effect on the expression and secretion of TolCV2 (Fig. 4C and D). Furthermore, we found that P4 mutation resulted in slight decrease in the transcriptional activities of *tolCV1* and *rtxA1* (Fig. 4E and F). Therefore, the TolCV1 mutant harboring mutations in NPPB binding residues (P4) had defect in exporting RtxA1 and bile salts. Further studies are required to explain why P4 mutation resulted in reduction of RTX toxin and TolC proteins. In this regard, we speculated that NPPB would enhance therapeutic efficacy of antibiotic treatment of *V. vulnificus* infections with dual action mechanisms: higher accumulation of antibiotics in bacterial cells and inhibition of the excretion of major cytotoxic factor RtxA1.

### NPPB inhibited *V. vulnificus*-mediated cytotoxicity

**Fig 5:**
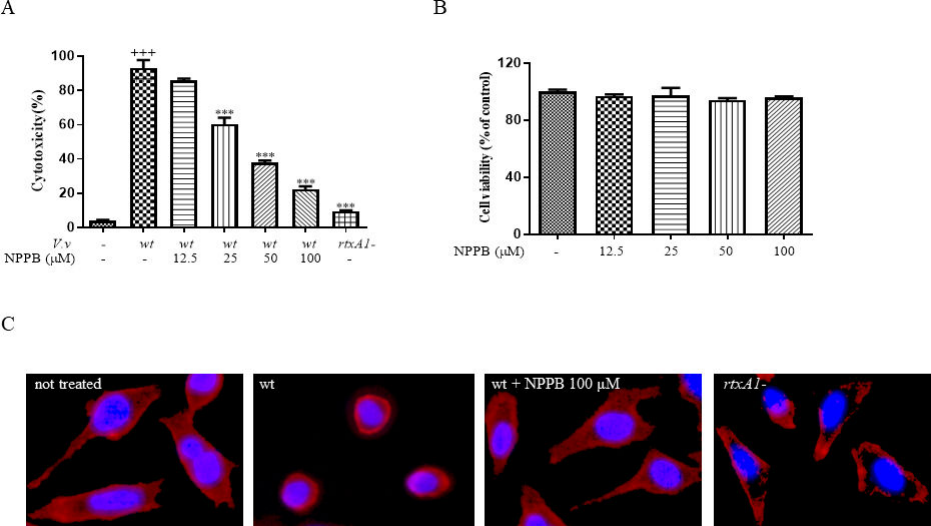
Effect of NPPB on *V. vulnificus*-induced cytotoxicity. (**A**) HeLa cells were pretreated with NPPB for 1 h before *V. vulnificus* infection at an MOI of 100 for 90 min. LDH in the supernatants was measured as a marker of cytotoxicity (^***^P < 0.001 vs *V. vulnificus* wild-type; ^+++^*P* < .001 vs untreated control). (**B**) HeLa cells were pretreated with NPPB for 24 h. Cell viability was detected by MTS assay. Absorbance was read at 490 nm (vs mock cells). Values are means ± SEM. (**C**) HeLa cells cultured in 8-well glass chamber plates were pretreated with NPPB for 1 h before *V. vulnificus* infection at an MOI of 100 for 90 min. The cells were stained with Alexa Fluor 594 wheat germ agglutinin (WGA, red) and Hoechst 33342 (blue) for labeling plasma membrane and nucleus, respectively. Images were acquired using a fluorescence microscopy. Abbreviations: *V.v*, *V. vulnificus*; wt, wild-type; *rtxA1*-, *rtxA1* mutant strain of MO6-24/O.

*V. vulnificus* rapidly kills host cells, and the RtxA1 toxin is the major player mediating the cytotoxicity (32, 38). Since NPPB significantly inhibited RtxA1 secretion, we conducted the LDH release assay to observe whether NPPB inhibits cytotoxicity of *V. vulnificus*. As shown in Fig. 5A, NPPB markedly suppressed the cytotoxicity of *V. vulnificus* wild-type to HeLa cells in a dose-dependent manner. To test whether NPPB itself would have any effect on host cell viability by inhibiting the chloride channel, we performed MTS assays. NPPB exhibited neither cytotoxicity nor growth-stimulating activity in host cells at tested concentrations, indicating that its inhibitory effect on cytotoxicity is mainly related to bacterial virulence (Fig. 5B). In addition, live cell staining was carried out to observe the effect of NPPB on the morphological changes of host cells infected with *V. vulnificus*. NPPB protected host cells from *V. vulnificus*-induced cell rounding, which was comparable to the infection by RtxA1 deletion mutant (Fig. 5C).

### NPPB increased the susceptibility of *V. vulnificus* to bile salts

**Fig 6:**
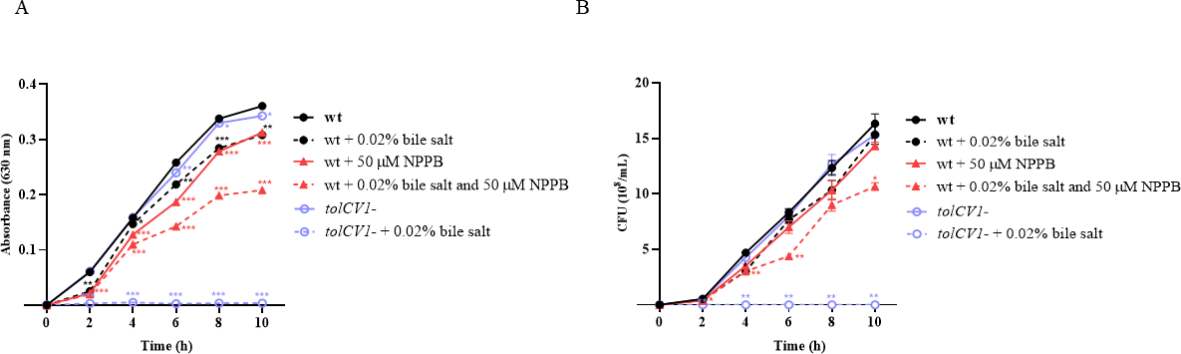
Effect of NPPB on the growth of *V. vulnificus* in the presence of bile salt. Overnight cultured *V. vulnificus* cells were diluted 1,000-fold with fresh LB broth. The diluted suspensions were cultured in 96-well microplates with or without 50 µM NPPB or/and 0.02% bile salt at 37°C. (**A**) The absorbance was read at 630 nm every 2 h. (**B**) *V. vulnificus* cultures were 10-fold serially diluted with PBS, the serial dilutions (10 µL) were loaded on LB agar plates overnight, and the numbers of grown colonies were counted. Results are representative of at least three independent experiments. Values are means ± SEM (vs. *V. vulnificus* wild-type in LB, ^*^P < 0.05, ^**^P < 0.01, ^***^P < 0.001). Abbreviations: *V.v*, *V. vulnificus*; wt, wild-type; *tolCV1*-, *tolCV1* mutant strain of MO6-24/O.

During the infections, *V. vulnificus* needs to survive in the gastrointestinal tract of the host. Large amounts of bile salts are distributed in the human intestinal environment (42). Our previous work showed that TolCV1 is crucial for *V. vulnificus* survival in the presence of bile salts (11). To study whether NPPB affects the susceptibility of *V. vulnificus* to bile salts, we tested the growth of *V. vulnificus* in the presence of bile salt and/or NPPB. The addition of 0.02% bile salt or 50 µM NPPB inhibited bacterial growth (Fig. 6A and B). The combination of NPPB and bile salt significantly suppressed the growth of *V. vulnificus* (Fig. 6A and B), suggesting that NPPB rendered *V. vulnificus* more susceptible to intestinal bile salts and would inhibit the intestinal growth and colonization of *V. vulnificus*.

### NPPB enhanced the therapeutic efficacy of tetracycline in *V. vulnificus*-infected mice

**Fig 7:**
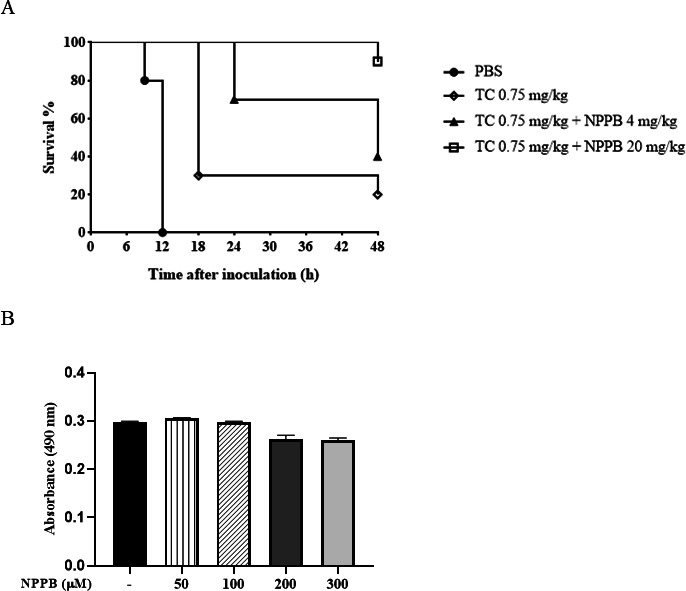
Effect of NPPB on *V. vulnificus*-induced lethality on mice. (**A**) Eight-week-old CD-1 mice were caged randomly in four groups of 10 mice each. Log phase *V. vulnificus* cells (2 × 10^6^ CFU/mouse) were administered to mice via the intraperitoneal route. One day or 2 h before *V. vulnificus* infection, mice were pretreated with NPPB or tetracycline twice via intraperitoneal injection. The infected mice were observed in the animal room for 48 h. (**B**) Mouse splenocytes were isolated from mouse spleen and treated with NPPB at different concentrations for 72 h. Cell viability was assayed using MTS assay. Absorbance was measured at 490 nm. Values are means ± SEM.

To study whether the NPPB-mediated TolCV1 depletion could potentiate antimicrobial therapy *in vivo*, we treated *V. vulnificus*-infected mice with NPPB in combination with tetracycline, which is recommended in clinics for the treatment of *V. vulnificus* septicemia (43). *V. vulnificus* wild-type cells (2 × 10^6^ CFU/mouse) were infected intraperitoneally to mice. Tetracycline and/or NPPB were injected twice, 1 day or 2 h before *V. vulnificus* infection. NPPB (4 or 20 mg/kg) significantly potentiated the therapeutic efficacy of tetracycline (0.75 mg/kg) (Fig. 7A). In the surviving animals, NPPB-treated mice suffered less, manifesting better fur condition and agile activity. We interpreted that the dual effects of NPPB on RtxA1 excretion and antibiotic efflux provided enhanced protection against *V. vulnificus* infections. To further confirm the safety of NPPB, we tested toxicity of NPPB in purified splenocytes and mice. The effects of NPPB on cell viability were evaluated via MTS assay. The mice splenocytes were treated with NPPB for 72 h. NPPB at the concentrations of 50 and 100 µM did not show any cytotoxicity on splenocytes (Fig. 7B). However, NPPB at high concentrations of 200 and 300 µM showed slight toxicity. Therefore, we set the maximum concentration of NPPB drug for our study to be 100 µM. The serum of the mice intraperitoneally administered NPPB for 3 days was isolated and whole-blood element analyzed to investigate the physiological effects of NPPB. The levels of ALT and AST, indicators of hepatotoxicity, were decreased compared with the non-treated group (Table 2). Creatinine levels, indicator of nephrotoxicity, did not show any difference between the two groups. In the case of BUN levels, a decrease was observed in the NPPB-treated group. As a result, the intraperitoneal injection of NPPB did not affect liver and kidney function in mice.

**TABLE 2:** Safety of NPPB on mouse liver and kidney^*a*^

NPPB	Liver function		Kidney function	
	AST (U/L)	ALT (U/L)	CRE (mg/dL)	BUN (mg/dL)
Non-treated	134.25 ± 22.48	50.25 ± 2.56	0.105 ± 0.002	26.3 ± 1.19
10 mg/kg	131.25 ± 38.09	44.25 ± 11.33	0.105 ± 0.002	22 ± 2.22

^*a*^ AST, aspartate transaminase; ALT, alanine transaminase; CRE, creatinine; BUN, blood urea nitrogen.

## DISCUSSION

The rapid emergence of multidrug-resistant *V. vulnificus* strains has posed a major challenge to the global healthcare system (44). Therefore, the development of antibiotic-enhancing drugs that combat drug resistance is the main goal of our current research. Recently, we investigated the functions of outer membrane protein TolCV1 in *V. vulnificus* and characterized it as an attractive target for treating *V. vulnificus* infections (11). In this study, we discovered that NPPB notably decreased the levels of TolCV1 protein in bacterial pellets (Fig. 1A). Therefore, we propose that NPPB could be considered as a potential antibiotic-enhancing drug against *V. vulnificus* infections. We found that TolCV1, a membrane protein, remained in the culture supernatant after NPPB treatment (Fig. 1A). This unexpected observation can be explained by the following potential mechanisms. NPPB might directly interact with TolCV1, altering its conformation in such a way that it remains solubilized even in the absence of detergents. It is possible that NPPB treatment induces partial solubilization of the membrane, creating a condition where some membrane-associated proteins, including TolCV1, are released into the supernatant.

LC/MS analysis showed that NPPB can directly bind with TolCV1 protein of *V. vulnificus* (Fig. 2A and B) and molecular docking study was carried out to predict the binding interactions between NPPB and *V. vulnificus* TolCV1. NPPB was predicted to form hydrogen bonds with several residues (ASN B:301, ASN B:305, PHE B:300) located inside of TolCV1 barrel-like structure (Fig. 2C). Biophysical methods such as isothermal titration calorimetry would provide a quantitative measurement of binding affinity and substantiate our claims. We constructed a *tolCV1* point mutant strain with alanine substitutions of four key amino acids (300, 301, 303, 305), the potential binding sites between *V. vulnificus* TolCV1 and NPPB predicted by molecular docking. We found that P4 mutant strain showed lower cytotoxicity than *V. vulnificus* wild-type strain (Fig. 2D) and the growth of the P4 mutant was greatly inhibited in the presence of bile salts (Fig. 2E and F), suggesting these four amino acids of TolCV1 play critical roles for TolCV1 to export RtxA1 toxin and bile salts.

The tripartite efflux pump is a major contributor to the transport of antibacterial drugs, which is important for bacterial survival especially during infections, where they contribute to multidrug resistance (10, 45). As a component of tripartite efflux pumps, TolCV1 is critical for exporting various toxic substances, antibiotics, and metabolites out of bacteria (7). In this context, we performed checkerboard assays to determine whether NPPB exhibits synergistic activities with antibiotics against *V. vulnificus* and observed that the addition of NPPB greatly enhanced the antibacterial effect of kanamycin, tetracycline, erythromycin, and ampicillin against *V. vulnificus* (Fig. 3). In addition, *V. vulnificus* TolCV1 was reported to be responsible for secreting RtxA toxin by participating in the assembly of T1SS (10, 46). *V. vulnificus* T1SS consists of ATPases RtxB and RtxE, a transmembrane linker RtxD, and the outer membrane porin TolC (47). Hence, we investigated the effect of NPPB on the synthesis and secretion of RtxA1 toxin in LB broth. To our surprise, NPPB increased RtxA1 remained in bacterial pellets, especially at a concentration of 100 µM, owing to its remarkable inhibition on the excretion of RtxA1 toxin in LB broth (Fig. 4A and B). In addition, we also investigated the effect of *tolCV1* point mutation on RtxA1, TolCV1 and TolCV2, and found that P4 mutant inhibited the secretion, expression, and transcriptional activities of RtxA1 and TolCV1 (Fig. 4C through F). Further investigation is warranted to study why *tolCV1* point mutation affect the expression of TolCV1 and RtxA1. Furthermore, NPPB considerably suppressed *V. vulnificus*-induced cytotoxicity and cell rounding to HeLa cells without affecting host cell viability (Fig. 5).

In the subsequent study, we examined the effect of NPPB on the susceptibility of *V. vulnificus* to bile salts. Bile salt acts as an antibacterial agent in the human intestine by disrupting bacterial membranes, denaturing proteins, chelating iron and calcium, and causing oxidative damage to DNA (48). Our previous work proved that TolCV1 is crucial for *V. vulnificus* growth in the environment with bile salt (11). The finding that NPPB significantly decreased TolCV1 proteins in bacterial pellets drove us to investigate whether NPPB affects the susceptibility of *V. vulnificus* to bile salts. We found that the combination of NPPB and bile salt significantly suppressed the growth of *V. vulnificus* wild-type (Fig. 6), suggesting that NPPB could greatly inhibit intestinal colonization during *V. vulnificus* infections. A previous study has identified that tetracycline has superior bactericidal activity against *V. vulnificus* (49). In this study, we evaluated the therapeutic efficacy of tetracycline alone or tetracycline in combination with NPPB in *V. vulnificus*-infected animals. Our *in vivo* studies indicate that the outcome of tetracycline plus NPPB therapy was better than that of tetracycline alone therapy in mice with *V. vulnificus* infections. NPPB enhanced the therapeutic efficacy of tetracycline in the *V. vulnificus*-infected CD-1 mouse model (Fig. 7A). Although the safety and efficacy of NPPB have been confirmed at the cellular and animal levels, further researches are needed for its clinical application.

In conclusion, these results indicate that NPPB can potentiate the activity of antibiotics against *V. vulnificus* infections through TolCV1 depletion, confirming its action as an efflux pump inhibitor. Furthermore, recent research has reported that the AcrAB-TolC efflux pump activity could preserve resistance acquisition by plasmid transfer in the presence of antibiotics in *E. coli* (50). Dead cells released the resistance-enhancing factor AcrA, a periplasmic component of the *E. coli* AcrAB-TolC efflux pump, that could interact with the TolC component on the outer membrane of live cells to stimulate multiple pathways to overcome antibiotic stress (51). Given these possibilities, further studies should focus on TolC as a new antibacterial therapeutic target and disclosing the molecular mechanisms underlying the action of antibiotic-enhancing effect of NPPB. This study was carried out with an idea that a specific inhibitor of *V. vulnificus* TolCV1 should have dual effects on virulence suppression and antibiotic susceptibility. Since the type 1 secretion systems of Gram-negative bacteria serve as the portal of virulence factor excretion and play a role in antibiotic resistance, we suggest that our study could be extended to multidrug-resistant cumbersome pathogens.

## SUPPLEMENTAL MATERIAL

Figure S1 aac.00502-24-s0001.docx NPPB LC-MS chromatogram.

ASM does not own the copyrights to Supplemental Material that may be linked to, or accessed through, an article. The authors have granted ASM a non-exclusive, world-wide license to publish the Supplemental Material files. Please contact the corresponding author directly for reuse.
