# Supplementary material for: TolCV1 inhibition by NPPB renders Vibrio vulnificus less virulent and more susceptible to antibiotics
Source: Antimicrob Agents Chemother. 2024 Dec 13;69(1):e00502-24. doi: 10.1128/aac.00502-24 (PMC11784226; doi:10.1128/aac.00502-24)

**Supporting Information**

**Figure S1.** (a) Standard NPPB LC-MS chromatogram at 360 nm (b) UV spectrum of the standard NPPB. (c) Mass spectrum of the standard NPPB. (d) Sample-WT+NPPB LC-MS chromatogram at 360 nm. NPPB was detected in the sample WT+NPPB. (e) UV spectrum of peak at 14.681 min in the (d). (f) Mass spectrum of peak at 14.681 min in the (d).


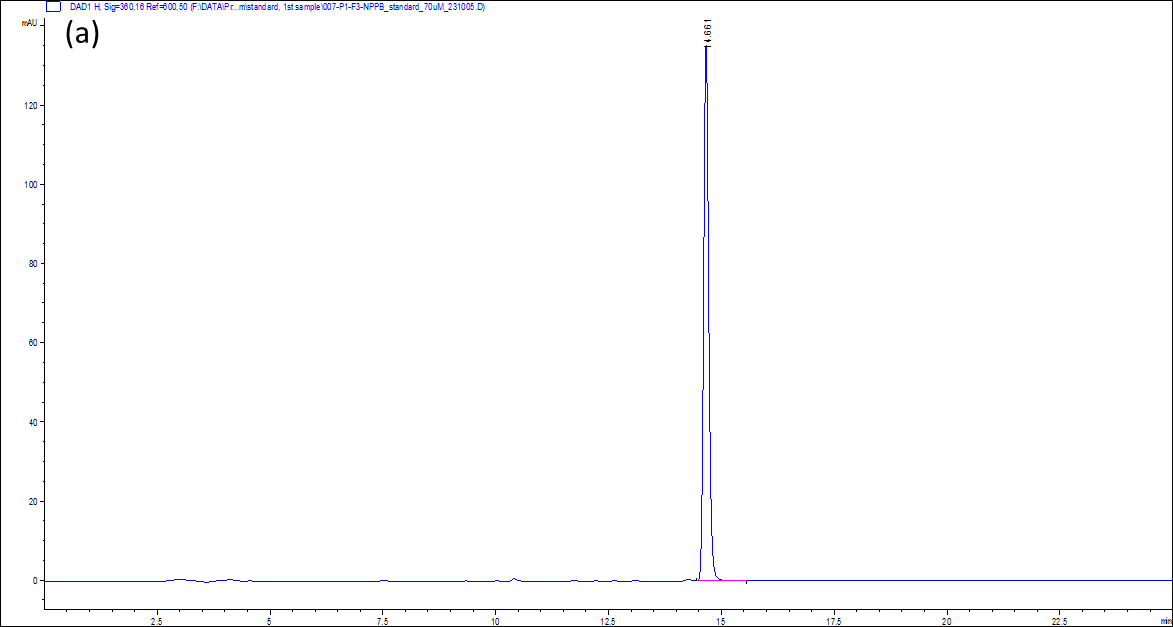


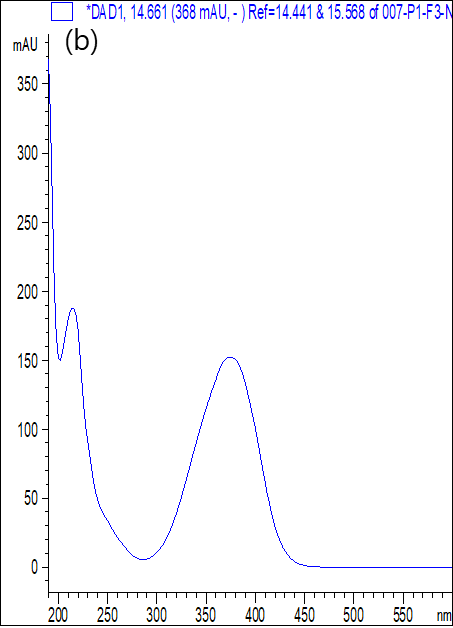

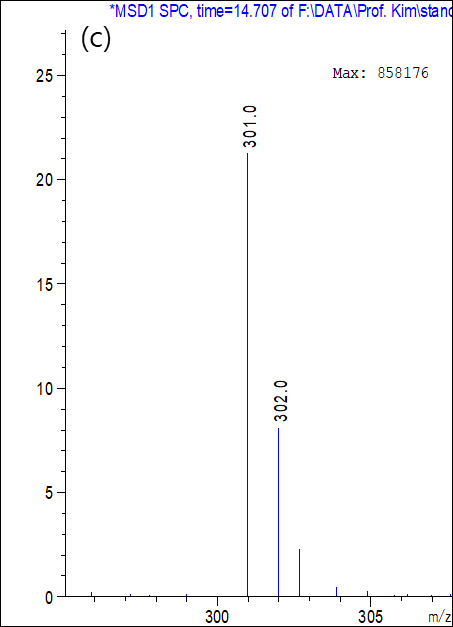


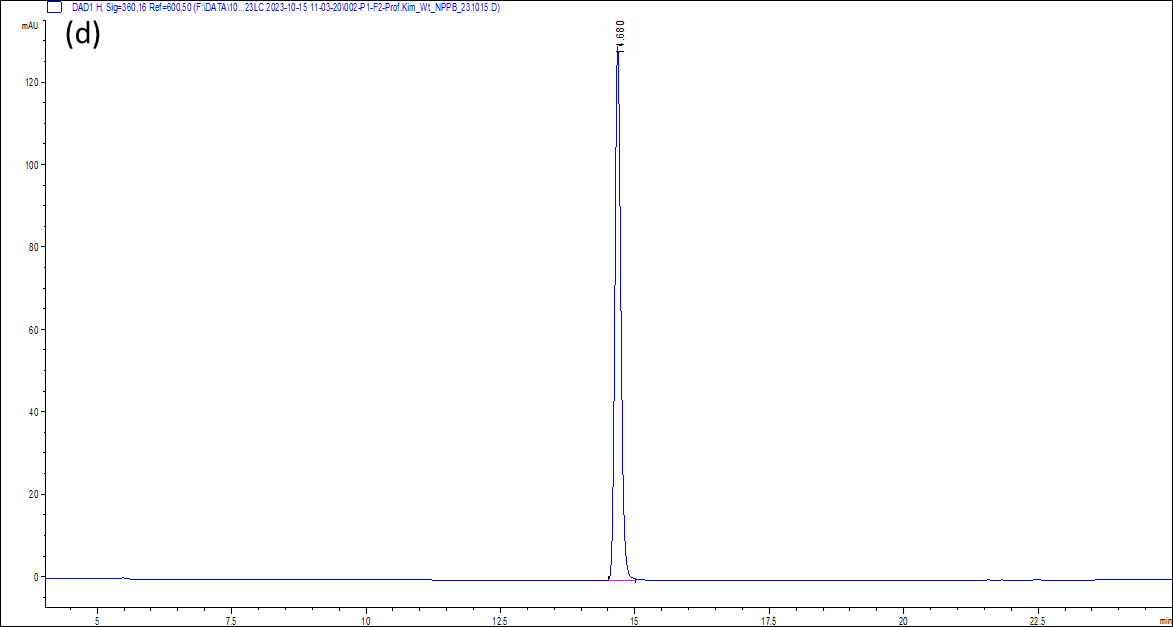


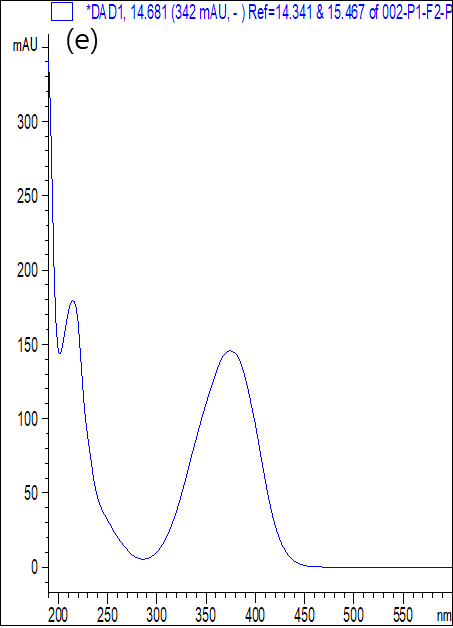

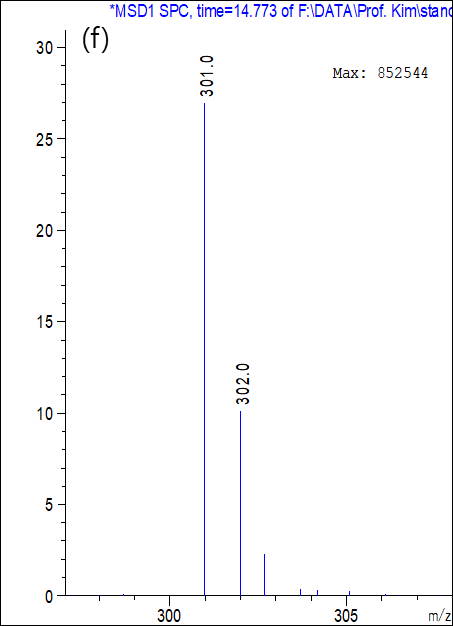

Supplement: Figure S1 — NPPB LC-MS chromatogram. [file aac.00502-24-s0001.docx]
